# Heart rate increase and inappropriate sinus tachycardia after cryoballoon pulmonary vein isolation for atrial fibrillation

**DOI:** 10.1007/s12471-021-01645-9

**Published:** 2021-11-11

**Authors:** C. van Deutekom, B. A. Mulder, H. F. Groenveld, R. G. Tieleman, A. C. P. Wiesfeld, E. S. Tan, I. C. van Gelder, M. Rienstra, Y. Blaauw

**Affiliations:** Department of Cardiology, University Medical Centre Groningen, University of Groningen, Groningen, The Netherlands

**Keywords:** Atrial fibrillation, Cryoballoon, Ablation, Pulmonary vein isolation, Heart rate

## Abstract

**Background:**

Cryoballoon pulmonary vein isolation (PVI) is a common therapy for atrial fibrillation (AF). While moderately increased sinus rhythm heart rate (HR) after PVI has been observed, inappropriate sinus tachycardia (IST) is a rare phenomenon. We aimed to investigate the prevalence and natural history of an abnormal sinus HR response after cryoballoon PVI.

**Methods:**

We included 169/646 (26.2%) patients with AF undergoing PVI with available Holter recordings before and 3, 6 and 12 months after the procedure. Patients with AF on Holter monitoring were excluded. Mean HR increase ≥ 20 bpm or an IST-like pattern (mean HR > 90 bpm or > 80 bpm when beta-blocking agents were used) following PVI was categorised as abnormal sinus HR response.

**Results:**

Following PVI, mean HR ± standard deviation increased in the entire group from 63.5 ± 8.4 to 69.1 ± 9.9 bpm at 3 months (*p* < 0.001), and to 71.9 ± 9.4 bpm at 6 months (*p* < 0.001). At 12 months, mean HR was 71.2 ± 10.1 bpm (*p* < 0.001). Only 7/169 patients (4.1%) met criteria for abnormal sinus HR response: mean HR was 61.9 ± 10.6 bpm (pre-ablation), 84.6 ± 9.8 bpm (3 months), 80.1 ± 6.5 bpm (6 months) and 76.3 ± 10.1 bpm (12 months). Even at 12 months, mean HR was significantly different from that pre-ablation in this group (*p* = 0.033). However, in patients meeting IST-like pattern criteria, mean HR at 12 months was no longer significantly different from that pre-ablation.

**Conclusion:**

Few patients had an abnormal sinus HR response after PVI. Peak HR was observed 3 months after PVI, but HR was still significantly increased 12 months post-ablation compared with pre-ablation. An IST-like pattern was rarely observed. In these patients, HR decreased to pre-ablation values within a year.

## What’s new?

- Previous studies have described an increased heart rate (HR) or inappropriate sinus tachycardia (IST) after pulmonary vein isolation (PVI) but have not reported long-term data on IST during follow-up.
- This is the first study to investigate the prevalence, time course and symptoms of an abnormal sinus HR response following cryoballoon PVI by combining patients with increased HR and/or an IST-like pattern.
- Abnormal sinus HR response was observed in few patients. In this group, mean HR was still significantly increased 12 months post-ablation compared with that pre-ablation. An IST-like pattern was rarely observed, and in these patients, HR normalised within a year.

## Introduction

Atrial fibrillation (AF) is the most common sustained cardiac arrhythmia with an estimated prevalence of 33.5 million in 2010 [1]. AF is associated with a higher risk of thromboembolic events, heart failure and death and has a significant impact on healthcare costs [2–4]. According to current guidelines, pulmonary vein isolation (PVI) can be considered as first-line treatment for patients with symptomatic AF [5]. PVI with the single-shot cryoballoon technique is a frequently used strategy [6]. It is a safe and effective procedure with known complications such as phrenic nerve palsy, access site complications, thromboembolic events and cardiac tamponade [7, 8].

Immediately following PVI, an increased sinus rhythm rate is observed [9–13]. In addition, during follow-up, patients may have elevated heart rates (HRs), and some fulfil criteria for inappropriate sinus tachycardia (IST) [14, 15]. Patients may experience symptoms of elevated HRs, and treatment may be troublesome.

The aim of the present study was to systematically investigate the prevalence and time course of an abnormal sinus HR response following cryoballoon AF ablation and its symptoms.

## Methods

### Study population

**Fig. 1:**
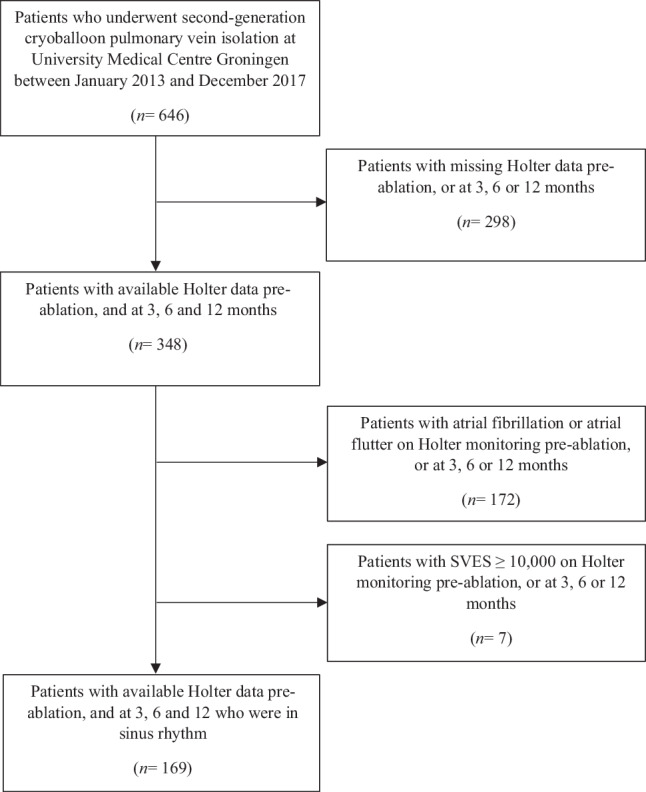
Flowchart of selection process study population. SVES supraventricular extrasystole

The study cohort consisted of 646 consecutive patients with AF who underwent cryoballoon PVI at the University Medical Centre Groningen between January 2013 and December 2017. Patients with missing Holter recordings pre-ablation or at 3, 6 or 12 months after the procedure were excluded (*n* = 298). In addition, patients with AF episodes, atrial flutter or supraventricular extrasystole ≥ 10,000 on one or more Holter recordings were excluded (*n* = 179). The remaining 169 individuals were included for analysis (Fig. 1). All data were retrospectively collected from patients’ medical files. All patients consented to the ablation procedure.

### Cryoballoon pulmonary vein isolation procedure

Our cryoballoon PVI procedure has been previously described [16]. Briefly, PVI was performed under conscious sedation. From 2014 and on, the 28-mm second-generation cryoballoon (Arctic Front Advance, CryoCath, Minneapolis, MN, USA) was used for ablation. In 2017, the approach proposed by Aryana et al. was adopted [17]. The number of cryoapplications was limited to once if time to isolation was < 60 s. A ‘bonus’ freeze of 120 s was delivered if time to isolation < 60 s was not observed.

### Clinical follow-up

Antiarrhythmic medication was continued for the first 3 months after the procedure, after which it was discontinued in patients without symptoms of AF. Scheduled outpatient clinic visits took place 3, 6 and 12 months after the procedure. These visits were preceded by 12-lead electrocardiography and 24-hour Holter monitoring. Medical history was acquired, and physical examination was carried out during these visits.

### Heart rate response

For each patient, mean 24-hour sinus HR was calculated based on Holter recordings pre-ablation and 3, 6 and 12 months after ablation. An abnormal sinus HR response was defined by combining the concepts of an increase in HR and the presence of IST-like pattern. We defined an increase in mean HR ≥ 20 bpm between the pre-ablation and the 3‑month Holter recording as abnormal. We defined an IST-like pattern as a mean HR > 90 bpm [18], or > 80 bpm if beta-blocking agents were used. A patient was labelled ‘IST’ if definition criteria were met at the 3‑month follow-up time point and not at the pre-ablation time point in combination with an increase in mean HR ≥ 10 bpm between the pre-ablation and 3‑month Holter recording. Group 1 included patients with a normal sinus HR response, and group 2 comprised patients with an abnormal sinus HR response.

### Outcomes

The main objective was to evaluate the prevalence and time course of abnormal sinus HR response and the prevalence of symptoms. Incidence of abnormal sinus HR response as defined above was selected as primary outcome for this study. Presence of symptoms was assessed based on reports by patients in their Holter diary or from the patient chart if symptoms were noted during outpatient clinic visits.

### Statistical analysis

Patient characteristics were compared between groups. Continuous variables are presented as mean ± standard deviation and categorical variables as number (percentage). A Chi-square test was used to compare categorical variables and a *t*-test or Mann-Whitney test to compare continuous variables depending on normality of the data. For differences in mean HR pre- and post-ablation, paired *t*-tests were performed. To assess a significant difference in mean HR between groups, *t*-tests for independent samples were performed, and a one-way ANOVA was used for > 2 groups. SPSS version 23.0 for Windows (IBM Corp, Chicago, IL, USA) was used to perform all statistical analyses. The significance level was set at *p* < 0.05 for all statistical tests.

## Results

### Patient characteristics

In total, 169 patients were included for analysis (Tab. 1). Of these 169 patients, 105 (62.1%) were male. Mean age was 58.8 ± 9.8 years, mean body mass index was 27.5 ± 4.7 kg/m^2^, and 137 patients (81.1%) had paroxysmal AF. In total, 69 patients (40.8%) had hypertension, 6 (3.6%) had heart failure, and 21 (12.4%) had coronary artery disease. Mean left ventricular ejection fraction was 54.4% ± 4.1, and 112 patients (66.3%) were on ≥ 1 antiarrhythmic drugs prior to PVI.

**Table 1:** Patient characteristics of research population, divided into research groups

Variable	Total population (*n* = 169)	Group 1 (*n* = 162)^a^	Group 2 (*n* = 7)^b^	*P*-value
Male sex	105 (62.1)	102 (63.0)	3 (42.9)	0.43
Age, years	58.8 ± 9.8	59.0 ± 9.4	52.2 ± 17.0	0.33
BMI, kg/m^2^	27.5 ± 4.7	27.5 ± 4.7	25.9 ± 4.8	0.35
*Type AF*				0.38
– Paroxysmal	137 (81.1)	130 (80.2)	7 (100)	
– Persistent	30 (17.8)	30 (18.5)	0	
– Long-term persistent	1 (0.6)	1 (0.6)	0	
Duration AF, days	2041 ± 1631	2082 ± 1643	1077 ± 946	1.00
Bundle branch block	11 (6.5)	11 (6.8)	0	1.00
Diabetes mellitus	10 (5.9)	10 (6.2)	0	1.00
Hypertension	69 (40.8)	68 (42.0)	1 (14.3)	0.24
Heart failure	6 (3.6)	6 (3.7)	0	1.00
Coronary artery disease	21 (12.4)	20 (12.3)	1 (14.3)	1.00
Peripheral artery disease	8 (4.7)	6 (3.7)	2 (28.6)	0.04
LVEF, %	54.4 ± 4.1	54.5 ± 4.0	52.9 ± 5.7	0.24
LAVI, mL/m^2^	33.1 ± 9.5	33.2 ± 9.6	30.0 ± 8.4	0.38
Prior antiarrhythmic drug use	112 (66.3)	108 (66.7)	4 (57.1)	0.69
Flecainide use	62 (36.7)	61 (37.7)	1 (14.3)	0.43
Sotalol use	39 (23.1)	37 (22.8)	2 (28.6)	0.66
Amiodarone use	18 (10.7)	17 (10.5)	1 (14.3)	0.55

Data are *n* (%) or mean ± standard deviation

*BMI* body mass index, *AF* atrial fibrillation, *LVEF* left ventricular ejection fraction, *LAVI* left atrial volume index

^a^ Group 1 comprises patients with normal heart rate (*HR*) response

^b^ Group 2 comprises patients with abnormal sinus HR response (HR increase ≥ 20 bpm and/or inappropriate sinus tachycardia)

### Time course of mean heart rate during follow-up

**Fig. 2:**
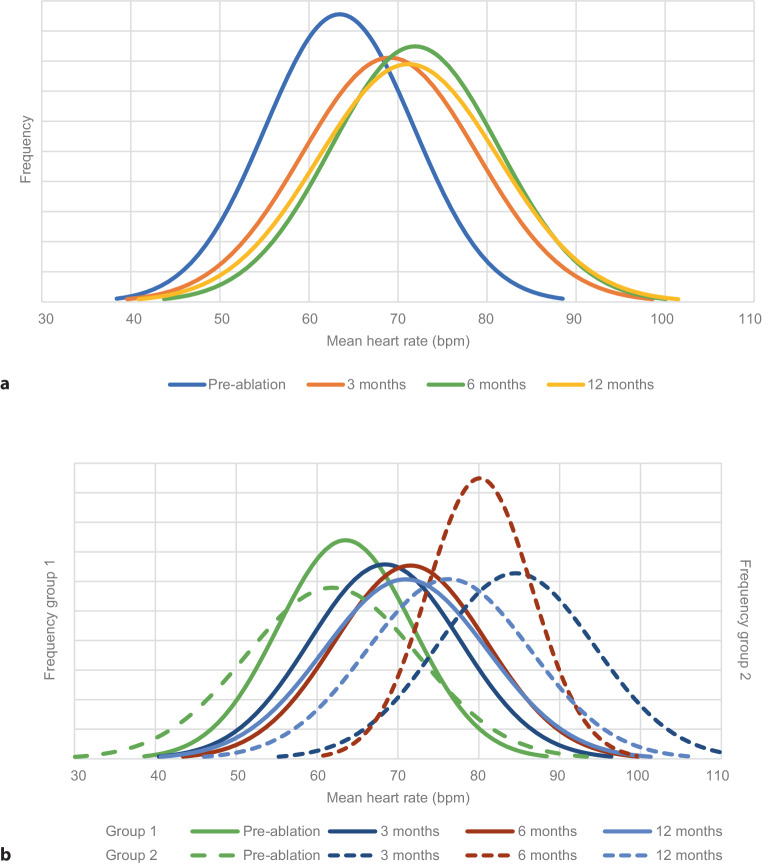
Distribution of mean heart rate pre-ablation and at 3, 6 and 12 months of follow-up. **a** Total study population. **b** Group 1 versus group 2

Fig. 2 shows the distribution of mean HR for the entire study population and per group pre-ablation, and at 3, 6 and 12 months post-ablation. A significant increase in mean HR was observed 3 months after ablation, and a further increase was observed at 6 months (Tab. 2). Mean HR decreased 12 months after PVI but was significantly increased compared with the pre-ablation time point (Tab. 2).

**Table 2:** Mean heart rate pre-ablation and during follow-up

Group	HR response	Pre-ablation	3 months	*P*-value	6 months	*P*-value	12 months	*P*-value
Overall		63.5 ± 8.4	69.1 ± 9.9	< 0.001	71.9 ± 9.4	< 0.001	71.2 ± 10.1	< 0.001
Group 1^a^		63.5 ± 8.3	68.4 ± 9.3	< 0.001	71.6 ± 9.4	< 0.001	71.0 ± 10.1	< 0.001
Group 2^b^	Abnormal sinus HR response	61.9 ± 10.6	84.6 ± 9.8	< 0.001	80.1 ± 6.5	0.007	76.3 ± 10.1	0.033
	HR increase ≥ 20 bpm	59.8 ± 10.0	83.2 ± 9.9	< 0.001	78.5 ± 5.2	0.017	75.5 ± 10.8	0.049
	IST	73.0 ± 1.0	94.0 ± 1.0	0.002	80.0 ± 8.7	0.261	76.7 ± 10.2	0.604

Data are mean heart rate ± standard deviation) in beats per minute. *P*-values are based on difference at follow-up compared with pre-ablation

^a^ Group 1 comprises patients with normal heart rate (*HR*) response

^b^ Group 2 comprises patients with abnormal sinus HR response (HR increase ≥ 20 bpm and/or inappropriate sinus tachycardia (*IST*))

Of the 169 patients, 6 (3.6%) met criteria for an HR increase ≥ 20 bpm. In this subgroup, mean HR at 12 months was significantly different from that pre-ablation (*p* = 0.049).

**Fig. 3:**
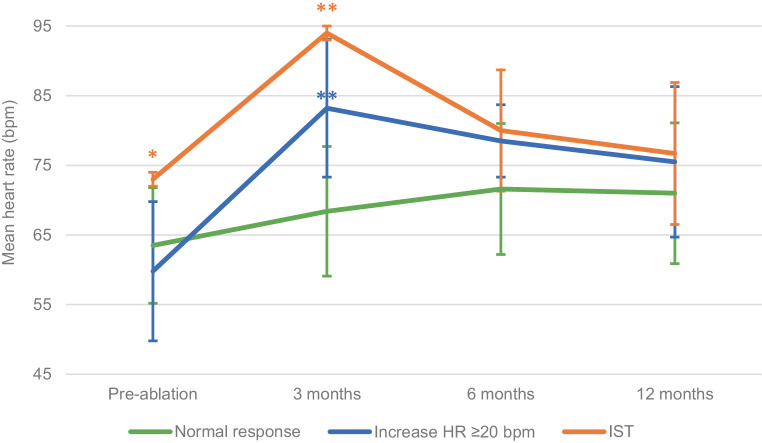
Mean heart rate pre-ablation and at 3, 6 and 12 months of follow-up per group. Significant differences in mean heart rate between groups are indicated (* compared with group ‘mean HR ≥ 20 bpm’; ** compared with group ‘normal response’)

Three patients (1.8%) met criteria for an IST-like pattern. Their mean HR increased by 21.0 ± 1.7 bpm between the pre-ablation and the 3‑month follow-up time points (Fig. 3). During follow-up, mean HR decreased to 80.0 ± 8.7 and 76.7 ± 10.2 bpm at 6 and 12 months, respectively. At 12 months post-ablation, mean HR was no longer significantly different from that pre-ablation.

Combining these concepts resulted in 7/169 patients (4.1%) meeting the definition of an abnormal sinus HR response. Mean HR increased from 61.9 ± 10.6 bpm pre-ablation to 84.6 ± 9.8 bpm 3 months after ablation (*p* < 0.001), after which the HR decreased to 80.1 ± 6.5 bpm at 6 months. At 12 months, mean HR was 76.3 ± 10.1 bpm and was still significantly different from that pre-ablation (*p* = 0.033). Tab. 3 shows medication use in these 7 patients during follow-up.

**Table 3:** Antiarrhythmic medication pre-ablation and during follow-up in abnormal sinus heart response group

Patient	Pre-ablation	3 months	6 months	12 months
1	Sotalol 80 mg BID	–	–	Bisoprolol 2.5 mg
2	Sotalol 80 mg BID	Verapamil 240 mg	Verapamil 120 mg	Verapamil 120 mg
3	Verapamil 40 mg TID	Verapamil 40 mg BID	Verapamil 40 mg BID	–
4	Sotalol 80 mg TID	–	–	–
5	–	–	–	–
6	–	–	Bisoprolol 5 mg	Bisoprolol 5 mg
7	Metoprolol 50 mg	Metoprolol 100 mg	Metoprolol 100 mg	–

*BID* two times daily, *TID* three times daily, – no antiarrhythmic medication

### Symptoms

In the group with an abnormal sinus HR response, 4/7 patients (57.1%) reported symptoms, consisting of feeling an increased HR in 2 patients (28.6%), palpitations in 2 patients (28.6%), a restless feeling in the chest in 1 patient (14.3%) and mild symptoms in 1 patient (14.3%). Splitting group 2 into ‘HR increase ≥ 20 bpm’ and ‘IST-like pattern’, we found that 4/6 patients (66.7%) in the group ‘HR increase ≥ 20 bpm’ reported symptoms, of whom 2 (33.3%) felt an increased HR, 2 (33.3%) had palpitations, 1 (16.6%) had a restless feeling in the chest and 1 (16.6%) experienced mild symptoms. In the IST group, 2/3 patients (66.7%) reported symptoms, of whom 1 (33.3%) felt an increased HR and 1 (33.3%) had mild symptoms.

Two of these patients required treatment: one received bisoprolol, which reduced symptoms, and the other underwent a concealed bypass tract ablation and was symptom-free afterwards.

## Discussion

The aim of this study was to determine the prevalence and time course of an abnormal sinus HR response after cryoballoon PVI. An abnormal sinus HR response was seen in 4.1% of the patients after cryoballoon ablation. One year after the procedure, this increase in HR was still present but not in the IST-like pattern group.

In this study, we used the combination of the presence of an IST-like pattern and an increase in HR as a concept for an abnormal sinus HR response. The occurrence of IST after cryoballoon PVI has also been described by others. In a small report, one of 66 patients developed IST, with an HR of 65 bpm pre-procedure and 100 bpm post-procedure [14]. Comparable results were described in a case report (HR pre-cryoballoon PVI 65 bpm, one month post-procedure 89 bpm) [15]. IST after PVI can occur regardless of the energy source. Pappone et al. reported on 24 patients who developed IST after radiofrequency PVI, which continued until one month after the procedure [9]. These observations are similar to the observations described in our study. However, the beforementioned papers did not report long-term data on IST or IST recovery during follow-up. Our data suggest that an IST-like pattern is rare but temporary since recovery of normal HR occurs within a year.

An increase in HR after cryoballoon PVI has been previously observed as well. Sakabe et al. investigated the cardiac autonomic nervous system (CANS) after cryoballoon PVI and found a significantly increased resting HR in 105 patients, from 58.9 ± 9.2 to 72.4 ± 9.5 bpm [12]. Furthermore, Miyazaki et al. aimed to evaluate CANS modulation, which generally leads to an increased HR, and its effect on cryoballoon PVI outcomes [11]. They observed an increase in HR in 69.5% of the patients, which returned to pre-ablation levels 6–12 months after PVI in 31.6% but remained increased at 12 months in 37.9%. They reported a mean HR increase of 18 bpm at 3 months, comparable to the 22.7-bpm increase in our study. There was no significant difference in patient characteristics between the groups with and without CANS modulation, which is comparable to our findings. Killu et al. included 1152 patients with radiofrequency PVI and reported a significant increase in HR, from 61 ± 11 to 76 ± 13 bpm post-ablation [19].

A possible explanation for the increase in HR is alteration of the CANS. This system consists of sympathetic, parasympathetic and sensory nerve fibres that arranged in so-called ganglionated plexi in the atrial epicardium [20, 21]. These plexi are mainly located around the antrum of the pulmonary veins, innervating one of the four pulmonary veins and the surrounding atrial myocardium [21–23]. The function of ganglionated plexi is to modulate interactions between the extrinsic autonomic nervous system and the CANS, which allows local regulation of the sinus rate [20, 23]. Because of their location, the major ganglionated plexi are affected by cryoballoon PVI. This treatment has been shown to elicit vagal responses, which were associated with modulation of the CANS [11, 24, 25]. This modulation resulted in reduction of parasympathetic activity, leading to an increase in HR. However, the effect may only last for 3 months after PVI [24]. This is in line with the peak in mean HR we saw at 3 months of follow-up.

In a study by Yu et al., 695 patients with paroxysmal AF underwent radiofrequency PVI, and 296 patients with persistent AF underwent radiofrequency PVI combined with a ‘Dallas lesion’ (which includes ablation of a roof line, posterior inferior line and anterior line) [26]. The authors observed a significant increase in mean HR at 3 months (from 68.8 ± 13.1 to 71.4 ± 0.7 bpm). They defined a group with high sinus rate after ablation (mean HR > 2 SD [≥ 92 bpm] on 24-hour Holter monitoring) and found that 36% maintained this higher HR 12 months after the procedure. Furthermore, there was no significant difference in the incidence of a high sinus rate after ablation between the PVI only group and the PVI plus Dallas lesion group.

Another prognostic parameter described in the literature is heart rate variability (HRV). Pappone et al. found that time and frequency domain HRV parameters decreased after PVI [9]. There was an increase of the low-frequency/high-frequency ratio from 1.09 ± 0.05 pre-ablation to 1.20 ± 0.07 one month post-ablation. This ratio remained elevated for 3 months (1.17 ± 0.06), returning to pre-ablation levels by 6 months (1.09 ± 0.05). Sakabe et al. reported a coefficient of variation of RR interval decrease from 2.36% ± 1.08 pre-ablation to 1.24% ± 0.68 one day post-ablation (*p* < 0.01).[12] In our study, we did not have data on HRV.

What does this imply for the role of CANS in AF and consequently PVI? Abnormal CANS functioning has been shown to contribute to the development of AF [21, 27]. Different mechanisms have been proposed, one of which is vagal stimulation. Vagal stimulation reduces the refractory period, increasing the risk for re-entry of signals and thereby increasing the inducibility of AF [28]. A different mechanism is through sympathetic activation, promoting ectopic activity by affecting depolarisation [29]. In addition, simultaneous activation of parasympathetic and sympathetic fibres has also been described as a trigger for AF [30].

However, with the current techniques, it is difficult to explore the exact role of the CANS in AF development. The role of the CANS in AF development together with the observation that cryoballoon PVI is associated with modulation of the CANS (leading to an increase in HR) may explain the association of an increase in HR after PVI with AF-free survival [19].

### Clinical implications

We observed a significant increase in HR after PVI. Several studies have found an association between an increase in HR and AF-free survival [10, 13, 19]. For our patients with an increased HR after PVI, this would imply a lower risk of AF recurrence compared with patients with no or a lower increase. As we only included selected patients without AF on their Holter recordings, we could not assess a relation with outcome or AF recurrence.

Additionally, our study has shown that patients may experience symptoms after PVI due to an abnormal sinus HR response, which was illustrated by continued use of medication. During outpatient clinic visits, physicians should reassure patients that these symptoms are related to the procedure and that they will subside over time. However, further exploration is needed to provide more insight into this topic.

### Strengths and limitations

Strengths of this study include the large initial cohort size and the amount of Holter data available for analyses.

A limitation is that only 7 patients met criteria for abnormal sinus HR response. This may have yielded insufficient data to identify any but the largest differences. Furthermore, to investigate symptoms related to an abnormal sinus HR response, we used patients’ self-reported data during Holter monitoring. These limitations complicated the ability to draw reliable conclusions on an abnormal sinus HR response and associated symptoms. An important prognostic parameter that would have been interesting to investigate, apart from mean HR, is HRV. However, data on HRV were not available for our study cohort.

## Conclusion

Abnormal sinus HR response after PVI was observed in 4.1% of the patients, with a peak mean HR at 3 months. Mean HR was still significantly increased at 12 months post-ablation compared with pre-ablation, except for in patients with an IST-like pattern, in whom the HR increase was temporary.
